# Analysis of the interaction of seeds with a centrifugal disk working body of a chamber-type treater

**DOI:** 10.1038/s41598-022-09204-x

**Published:** 2022-03-30

**Authors:** Mayya Sukhanova, Salavat Mudarisov, Victor Zabrodin, Andrey Bondarev, Faile Gallyamov

**Affiliations:** 1 Department of Technical Mechanics and Physics, Azov Black Sea State Engineering Institute FSBE HPE “Don State Agrarian University” in Zernograd, Zernograd, Russian Federation; 2 Department of Agricultural and Technological Machines, Federal State Budgetary Educational Establishment of Higher Education “Bashkir State Agrarian University”, Ufa, Russian Federation; 3 Department of Road Construction, Communal and Agricultural Machines, Federal State Budgetary Educational Establishment of Higher Education “Bashkir State Agrarian University”, Ufa, Russian Federation

**Keywords:** Electrical and electronic engineering, Mechanical engineering

## Abstract

Presowing seed treatment is the first step in growing crops and obtaining a predicted high-quality yield. It plays a vital role in an integrated plant protection system. When treating seeds before sowing, it is crucial to avoid seed damage by the machine working bodies to obtain the planned harvest. For that reason, when designing new machines, it is necessary to exclude the use of auger working bodies and reduce the treatment speed. This paper analyzes the interaction of seeds with the working surfaces of centrifugal disk distribution bodies of pressown seed treatment machines. This paper considers the influence of geometric and kinematic factors on the distribution of particles at the outlet of the disk working body. Theoretical dependences that allow determining the relative speed of seed escape from the blades of a centrifugal disk have been obtained. An experimental plant allowing testing of the effectiveness of the proposed technical solution has been designed and manufactured. Theoretical and experimental studies allowed us to determine the conditions necessary for uniform chemical treatment of seeds and reduce the impact of centrifugal-type working bodies on seeds when treated with chemical or biological preparations before sowing.

## Introduction

For the steady growth of crop production to keep up and increase soil fertility, it is necessary to improve existing technologies and master new environmentally safe methods of growing crops^1–6^. Presowing seed treatment is the most significant activity in crop growth and cannot be ignored since it plays a crucial role in sustainable crop production^7–13^.

The requirements for the quality of agricultural products increase, thus increasing the requirements for agricultural production technologies. Therefore, the technical means that ensure the implementation of agricultural operations need improvement. For the effective presowing treatment of seeds with chemical and biological preparations, it is necessary to use technical equipment with working bodies to treat the seeds effectively and perform it as carefully as possible, excluding the loss of seeds caused by their damage^14–19^. Seed damage by machine working bodies is known to negatively affect seed sowing properties, yield and germination^14,18–20^. Existing technical equipment hinders the effective introduction of progressive seed treatment methods and obtains high yields by the increased seed damage rate of grain and leguminous crops. Reduction in yields resulting from seed damage reaches 20% or more^21^. According to scientists, seed material damage reduces wheat and barley yield by 2–3 times. In some cases, the yield falls by 5^21^. 20–25% of damaged seeds lead to a decrease in yield by more than 0.3 t/ha^21^. According to scientists, the greatest damage to the seed material is observed when interacting with the working bodies of grain blowers and when using a machine with a disk working body of the PS-10 type^17^ for presowing seed treatment. For preserving the seeds, the working bodies of the machines for presowing treatment should have a minimal traumatic effect on the seeds. To modernize existing machines and create new, more advanced configurations, the traumatic effect of the working bodies of existing devices on seeds needs analysis. In addition, the design parameters and kinematic modes of operation of machines minimizing seed damage are required.

Machines for presenting seed treatment with chemical and biological preparations differ in the working body design. Thus, there are mass-produced drum-type, chamber- and auger-type treaters. The main advantage of chamber treaters is the ability to regulate the flow of the preparation when sprayed in the chamber, which allows reducing the flow of working fluid up to 25–50%. However, a severe disadvantage of chamber-type devices is seed damage when they hit against the treater chamber working surfaces under the action of centrifugal force of the rotating feeding disk. The analysis of technological processes of continuous and cyclical treaters proves that the seed damage caused by centrifugal distributors is 3.5–4.8%^15^. The works of scientists devoted to the study of centrifugal devices allow us to conclude that the radius of the outer edge of the blades (the disk radius) and the disk angular velocity have the most significant influence on the relative speed of the particle exit^22,23^. At the same time, the seed damage degree increases with an increase in the angular speed of the disk rotation.

This study aims to analyze the interaction of seeds with the working surfaces of centrifugal disk devices to justify the conditions for the uniform covering of seeds with the preparation and reduce seed damage.

The following research tasks need solutions for achieving the target goal:

- determining the factors affecting the uniformity of seed treatment and their damage by working disk bodies;
- developing a mechanical and mathematical model of the interaction of seeds with a centrifugal disk working body of chamber-type treaters;
- Determine the design parameters and kinematic modes reducing the seed damage by a centrifugal disk working body;
- an experimental verification of the obtained theoretical dependencies.

## Materials and methods

There are several factors affecting seed damage. When studying the process of seed interaction with the disk working body of the chamber treater, the seeds are considered loose particles. The technological process of interaction of the centrifugal working body with the loose particles includes the following phases:

- the supply of particles to the working area of the distributing disk;
- movement of particles over the disk working surfaces;
- interaction of particles with reflective surfaces.

Studies on centrifugal disk distributors^11,22,23^ show that when organizing the place where particles are fed to the disk blades, it is necessary to take into account:

- the distance from the axis of rotation to the center of the feed zone;
- the shape of the longitudinal and cross-section of the blades;
- the disk rotation speed;
- the seed feed velocity to the disk blades;
- the direction of the feed velocity vector.

**Figure 1:**
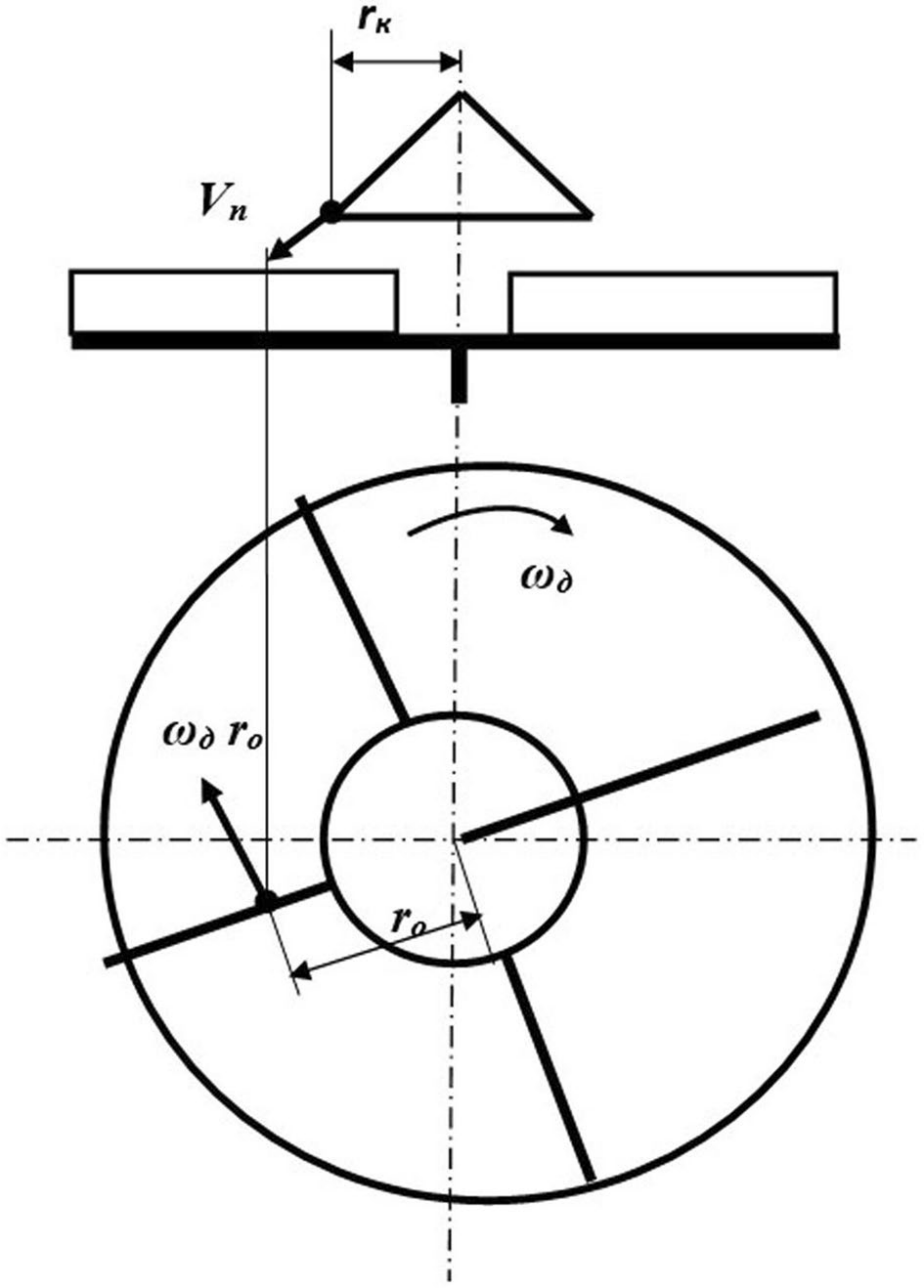
Analysis of the process of interaction of the seeds with the blades of the centrifugal disk. *Source:* author's development.

These factors influence the distribution of particles at the outlet of the centrifugal disk working body of the chamber-type etcher (Fig. 1).

**Figure 2:**
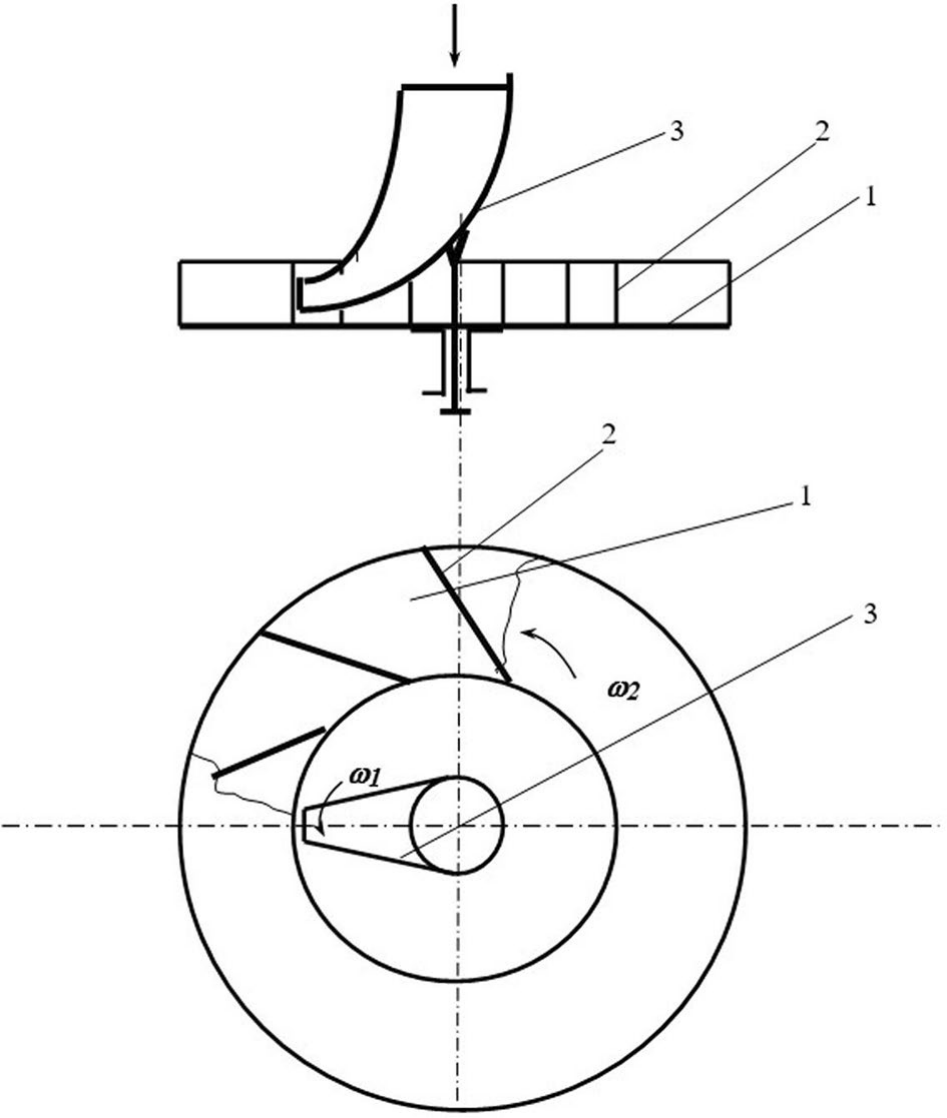
Centrifugal distributor of the presowing treatment. *Source:* author's development.

In seed treaters equipped with a centrifugal disk working body, the particles should be distributed along the angle of the sowing sector. Theoretically, central loading can provide a uniform distribution of particles in the sowing sector^22^. However, slight deviations of the feed zone center from the disk rotation axis violate the uniform distribution law. The curve of the particle distribution at the outlet of the director cone becomes convex, close to the standard distribution curve. Seeds are subject to a frontal (direct) impact when they fall on the working surface of the blades at a right angle. This method of feeding leads to a stronger impact interaction of particles with the blades (Fig. 1). To avoid this phenomenon, director cones are used in some device designs^22^. When using director cones, the particles enter the blades of the disk at a distance equal to the outer radius of the cone (Fig. 2).

The impact interaction velocity depends on the disk rotation angular velocity, the outer radius of the funnel-shaped director cone, the velocity of the particles escaping from the cone and the direction of the particle velocity vector. When the directions of the particle feed velocity and the rotary disk velocity vectors do not coincide, seeds are covered with the preparation unevenly and can be damaged.

Consider a disk centrifugal working body to determine the possibility of implementing the law of uniform density of particle distribution at the outlet of the apparatus (Fig. 2). The centrifugal working body consisted of centrifugal disk 1 with blades 2 and guide funnel 3. Guide funnel 3 is installed coaxially with disk 1 and can rotate with an angular velocity *of ω*_*1*_. Disk 1rotates with an angular velocity *of ω*_*2*_*.* The rotation directions of disk 1 and funnel 2 can coincide or be opposite.

**Figure 3:**
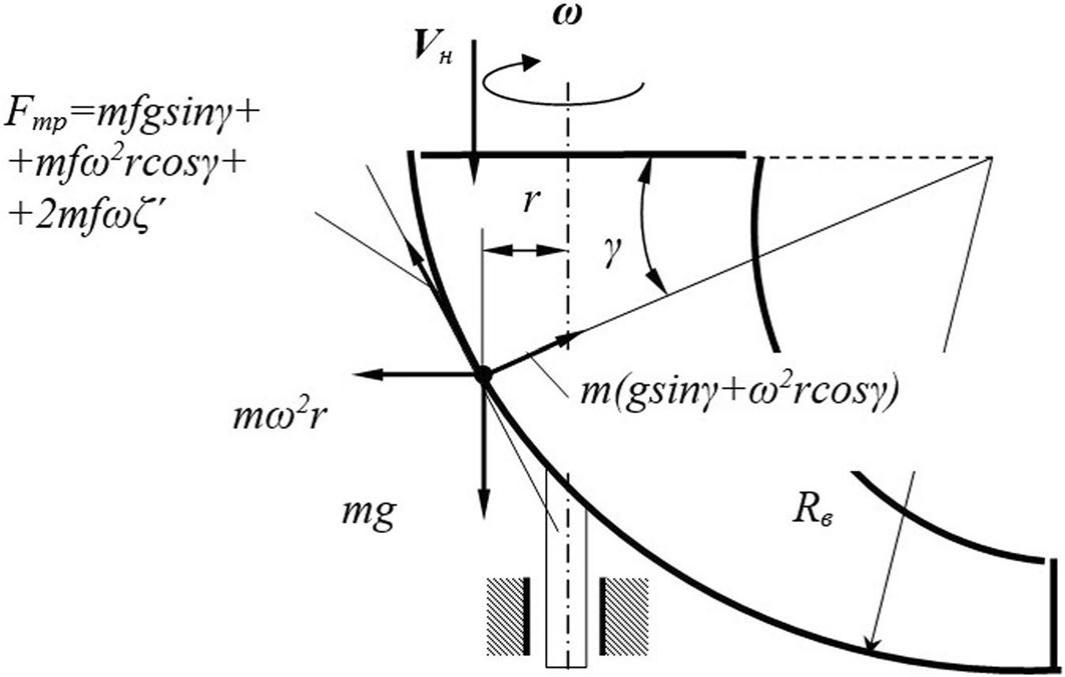
Analysis of the particle motion over the working surface of the director cone. *Source:* author's development.

Consider the movement of a particle along the inner surface of the guide funnel to justify the working body parameters (Fig. 3).

Write the differential equation of the particle motion:

1 $$ {\text{x}} = g\cos\gamma - {2}f\omega {\text{x}} - \omega {2}r\sin\gamma - fg\sin\gamma - f\omega {2}r\cos\gamma $$

where *x is* the distance the particle covers when going along the guide funnel and is related to the angle *γ* by the expression *γ* = *x/R*_*in*_; *ω* is the angular velocity of the director cone rotation.

The distance *r* from the rotation axis to the point is related to the diameter *d *_*of*_ the funnel upper part and its parameters by the expression:

2 $$ r = \frac{{d_{{\text{B}}} }}{2} - R_{{\text{B}}} \left( {1 - \cos\gamma } \right). $$

An approximate solution of the differential equation can be performed using one of the numerical methods, for which purpose the differential Eq. (1) is represented as a system:

3 $$ \left\{ {\begin{array}{*{20}c} {\xi^{\prime} = V_{r} } \\ {V_{r}^{/} = \omega^{2} \left[ {\frac{g}{{\omega^{2} }}\cos\gamma - f\left( {\frac{{2V_{r} }}{\omega } + r\cos\gamma + \frac{g}{{\omega^{2} }}\sin\gamma } \right) - r\sin\gamma } \right]} \\ \end{array} } \right. $$

**Figure 4:**
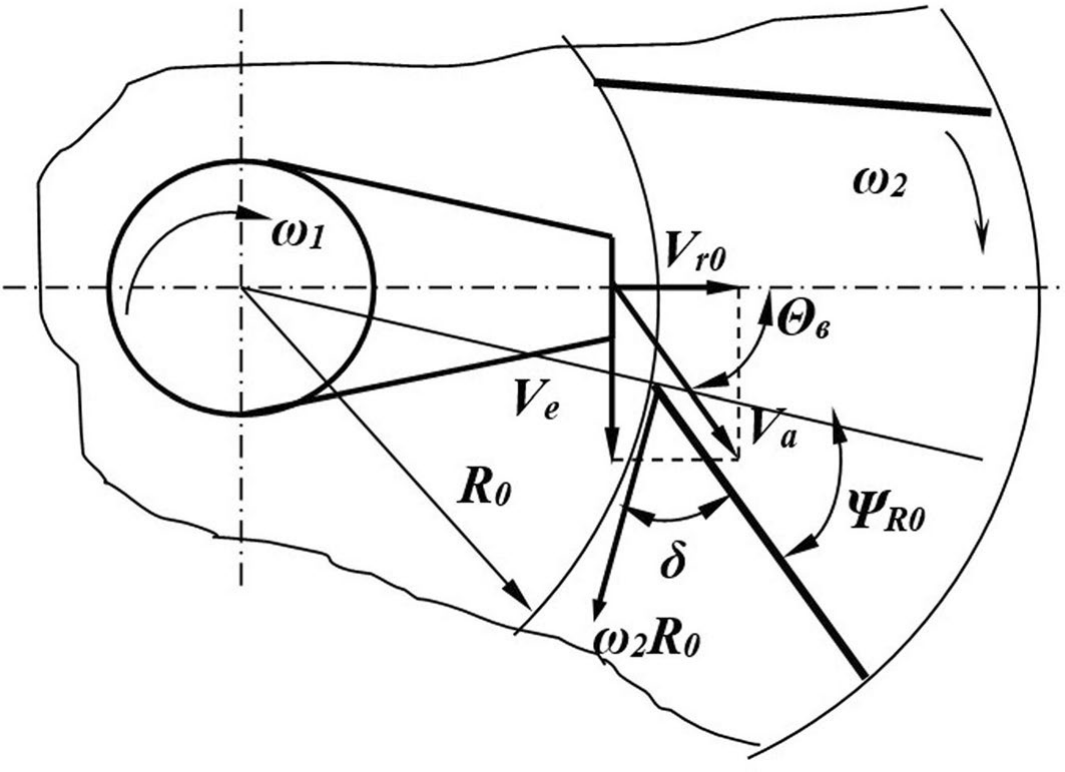
Determining the angle of the blade setting. *Source:* author's development.

The solution of equation system (3) (initial data are *t* = *0 ζ* = *0, V*_*r*_ = *V*_*h*_) helps determine the relative velocity *V*_*r0*_ of the particle exit from the director cone (Fig. 4).

The absolute particle velocity is determined from the geometric sum of vectors of relative *V*_*r0*_ and drift*V*_*e*_ = *ωr*_*max*_ velocities. The distance *r*_*max*_ is determined from the expression *r*_*max*_ = *R*_*in*_(1 − *siny*_*max*_) − *(d*_*in*_*/2)*.

The particles will leave the funnel at an angle *θ*_*b*_ (Fig. 4) equal to

4 $$ \theta_{{\text{B}}} = arctg\left( {\frac{{\omega r_{{{\text{max}}}} }}{{V_{r0} }}} \right) $$

To reduce the hit force of particles leaving the director cone against the blades of the centrifugal disk, it is necessary to create a condition under which the direction of the velocity vector *V*_*a*_ is close to the angle *в*_*b*_, that is, *δ* = *(π/2)-*_*R R0*_ = *в*_*b*_.

From the condition of equality of angles, we obtain:

5 $$ \psi_{R0} = \frac{\pi }{2} - arctg\left( {\frac{{\omega \,r_{{{\text{max}}}} }}{{V_{ro} }}} \right) $$

From the analysis of the forces acting on the particle and the differential equation solution, the relative velocity at the moment of the particle's exit from the blade is equal to:

6 $$ V_{r2} = \frac{{\omega_{2} \left[ {R_{2} \cos\left( {\phi + \psi_{Ro} } \right) + l_{2} \cos\phi } \right] \cdot \left[ {R_{0} \omega_{2} \cos\left( {\phi + \psi_{Ro} } \right) + V_{{\text{H}}} \left( {1 - \sin\phi } \right)} \right]}}{{\omega_{2} R_{0} \left( {1 + \sin\phi } \right)\cos\left( {\phi + \psi_{Ro} } \right) + V_{{\text{H}}} \cos^{2} \phi }} $$

where *R*_0_ and *R*_2_ are the radii of the inner and outer edges of the blades; *ω*_2_ is the angular disk velocity; *ψ*_*R*0_ is the angle of the blade inclination to the disk radius; *l*_2_ is the working length of the blade; *φ* is the angle of friction of the particle on the disk blades; and *V*_*h*_ is the initial velocity of the particle along the blade.

The obtained dependence (Formula 6) shows the dependence of the relative speed of the seed descent from the blades of the centrifugal disk on the design (radii *R*_*0*_ and *R*_*2*_, the length of the working part of the blade *l*_*2*_ and the angles of setting of the blades to the disk radius *ψ*_*R0*_) and kinematic (angular velocity *ω*_*2*_ and the projection of the vector of initial velocity on the blade plane *V*_*n*_) parameters and the friction angles of a particle on the disk blades.

Studies on centrifugal devices allow us to conclude that the radius of the outer edge of the blades (the disk radius) and the disk's angular velocity have the most significant influence on the relative speed of the particle exit^22^. At the same time, the seed damage degree increases with an increase in the angular speed of the disk rotation.

Seed damage is significantly affected by the direction of disk rotation relative to the director cone.

**Figure 5:**
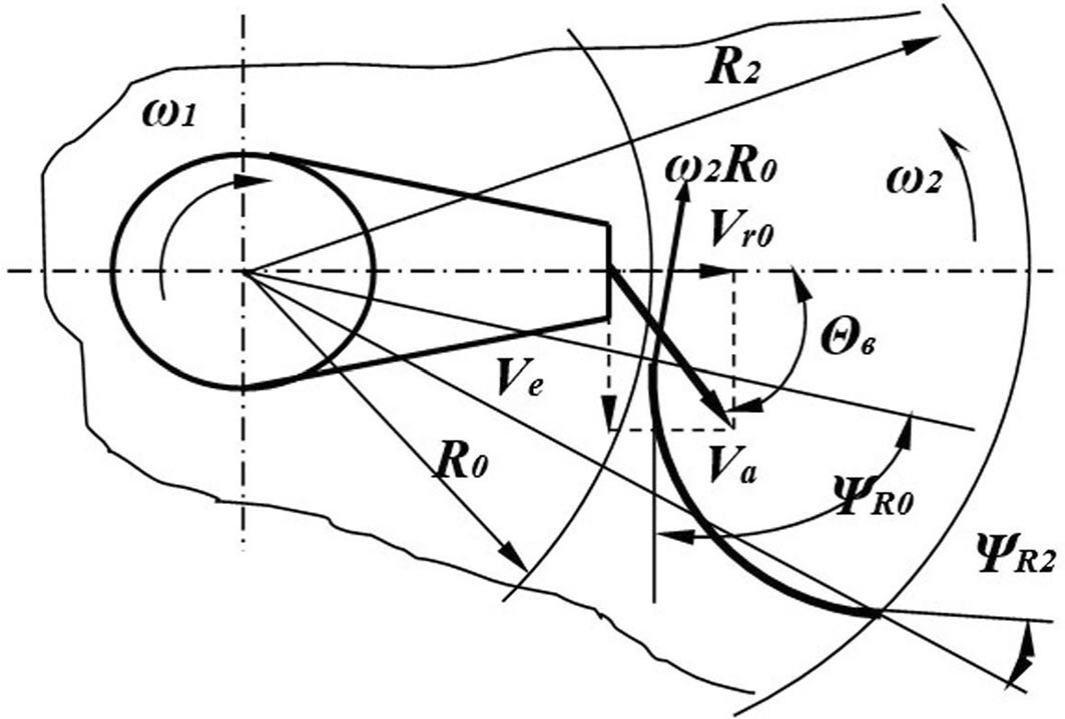
Determine the angle of the blade setting in the case of counterrotation. *Source:* author's development.

In the case of the counterrotation of the director cone and the centrifugal disk, curved blades are better for installation (Fig. 5) with an angle *ψ*_*Ro*_ of setting at the inner edge close to the angle given in Formula 5.

If the generatic line of the blade is made in the form of a circle with a radius *r*_l_, as shown in Fig. 5, then the angle of the blade setting β_l_ (Fig. 5) is determined from the formula:

7 $$ \beta_{\uppi } = 2 \cdot arctg \cdot \frac{{R_{2} \cdot \sin\psi_{R2} - R_{0} \cdot \sin\psi_{Ro} }}{{R_{2} \cdot \cos\psi_{Ro} + R_{0} \cdot \cos\psi_{Ro} }}, $$

**Figure 6:**
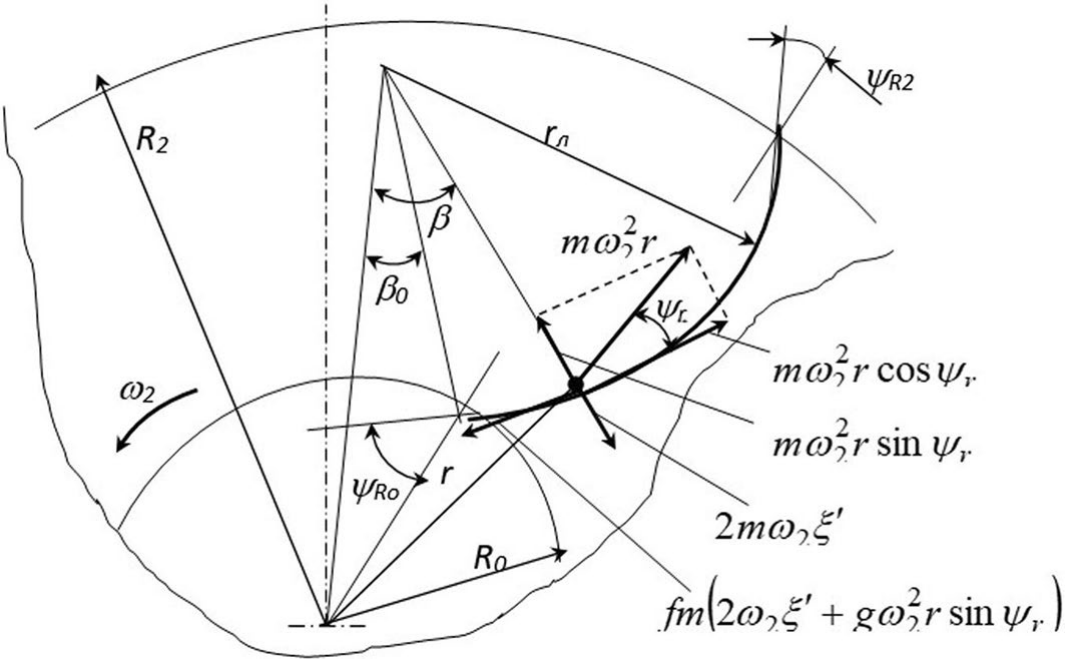
Diagram of the forces acting on the particle when moving along the curved blade. *Source:* author's development.

Figure 6 shows the forces acting on a particle moving along the blade. By considering these forces, the differential equation of the particle motion is obtained:

8 $$ {\ddot{\text{x}}} = \omega_{2}^{2} \left[ {c \cdot \sin\beta - f\left( {\frac{{2V_{r} }}{{\omega_{2} }} - c \cdot \cos\beta + r_{\uppi } + \frac{g}{{\omega_{2}^{2} }}} \right)} \right], $$

where $$ c = \sqrt {r_{\uppi }^{2} + R_{0}^{2} - 2r_{\uppi } R_{0} \sin\psi_{Ro} } ; \;\beta = \frac{\xi }{{r_{\uppi } }} + \beta_{0}$$; $${ }\beta_{0} = {\text{arcsin}}\frac{{R_{0} \cos\psi_{Ro} }}{c}$$.

The first-order derivative of the particle tracking along the guide funnel is the relative velocity of the particle movement:

9 $$ \left\{ {\begin{array}{*{20}c} {{\dot{\text{x}}} = V_{r} } \\ {V_{r}^{{\prime }} = \omega_{2}^{2} \cdot \left[ {c \cdot \sin\beta - f \cdot \left( {\frac{{2V_{r} }}{{\omega_{2} }} - c \cdot \cos\beta + r_{\uppi } + \frac{g}{{\omega_{2}^{2} }}} \right)} \right].} \\ \end{array} } \right. $$

The solution of the resulting equation allows determining the duration of the particle staying on the blade and the speed of its departure.

**Figure 7:**
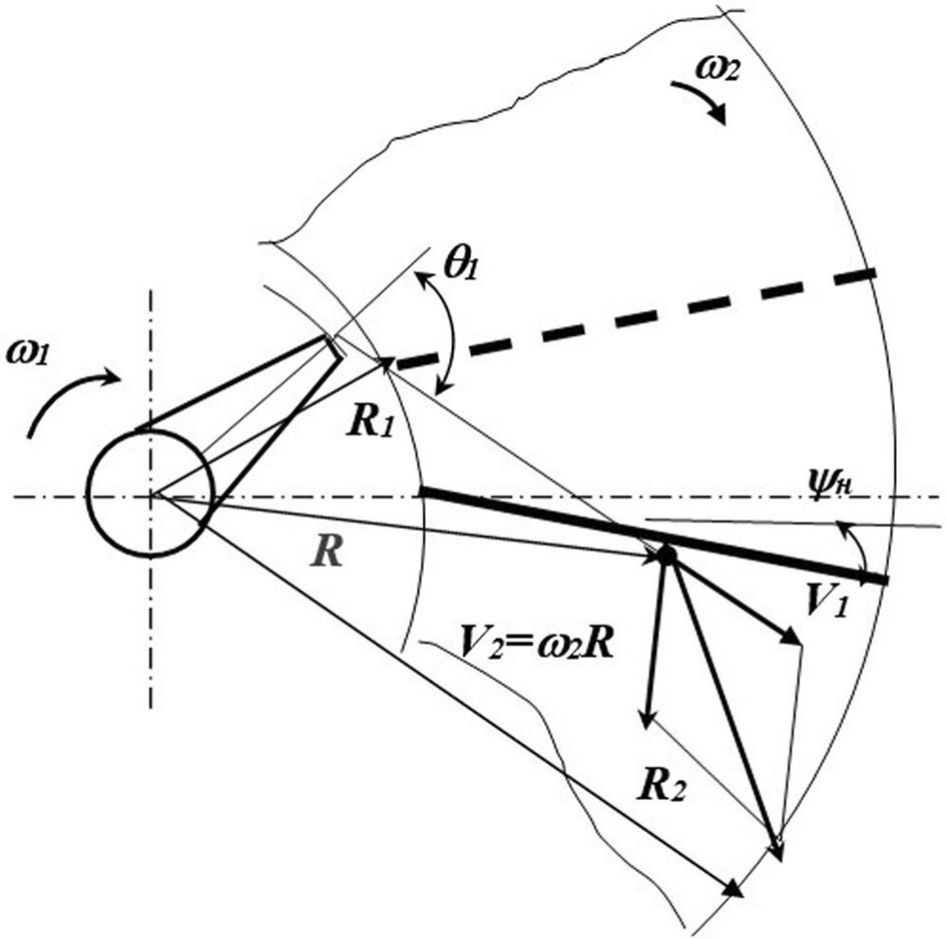
Diagram of the interaction of seeds with the blades of a centrifugal disk. *Source:* author's development.

To evenly cover the seeds with the preparation, the particle should be picked up by the blade on the fly. Analyzing the interaction of the particle with the blade of the outer disk (Fig. 7), determine the radius of the most distant point from the axis of the disk rotation.

During the flight time, *t* in the seed will move in the interblade space in the direction of the vector *V*_*1*_ by a distance *V*_*1*_^*.*^*t*_*in*_ Subsequently,

10 $$ R = \sqrt {R_{1}^{2} + \left( {V_{1} \cdot t_{{\text{B}}} } \right)^{2} + 2R_{1} \cdot V_{1} \cdot t_{{\text{B}}} {\text{c}}os\theta_{1} } $$

The disk rotation angle during time *t*_*в*_ is related to the angle *2π/z*_*2*_ between adjacent blades by the dependence $\alpha_{{\text{H}}} = \frac{2\pi }{{z_{2} }} - \omega_{2} t_{{\text{B}}}$.

Then, time *t*_*в*_ is determined from the expression:

11 $$ t_{{\text{B}}} = \frac{{2R_{0} \cos\frac{{\alpha_{{\text{H}}} }}{2}\cos\left( {\frac{{\alpha_{{\text{H}}} }}{2} - \psi_{Ro} } \right)}}{{V_{1} \sin\left( {\theta_{1} - \alpha_{{\text{H}}} } \right)}} $$

By substituting the value of time t in Formula (9), it is possible to determine the radius *Rmax* of the most distant point of the seed meeting with the blade. The interaction of the seeds with the blade of the centrifugal disk will occur in the area from *R*=*R1* to *R*=*Rmax*.

An experimental plant allowing testing of the effectiveness of the proposed technical solution has been designed and manufactured.

**Figure 8:**
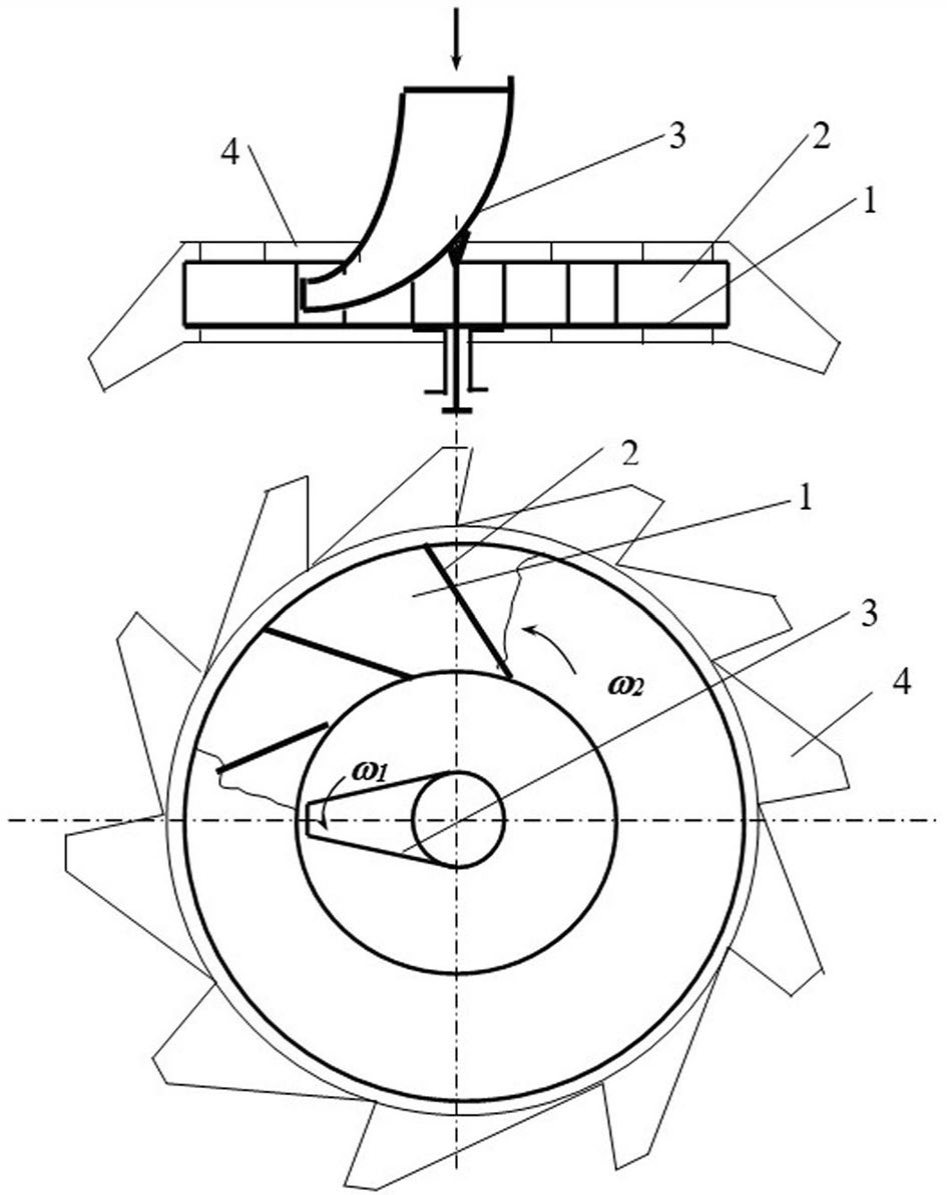
Scheme of the experimental plant. *Source:* author's development.

The plant consists of centrifugal disk 1 with rectilinear blades 2 and funnel 3. Around the centrifugal disk, there is a catch mechanism 4, divided by baffles into 12 compartments. Each compartment covers a central corner of 30° (Fig. 8).

The drive of the director cone and the centrifugal disk is independent. It allows changing the frequency and direction of the centrifugal disk rotation.

The studies were carried out at the moment when the disk and the director cone rotations coincided, and they rotated toward each other. . The experimental unit has a trap separated by partitions to assess the quality of seed distribution on the disk.

Particles of two grains of different sizes (winter wheat and sorghum) were used to make the research results more meaningful. Wheat was chosen for research since it is the most popular type of agricultural crops cultivated in the world. Sorghum occupies 70–75 million hectares in world agriculture. Its acreage ranks fifth after wheat, rice, corn and barley^24–26^. Feed qualities of sorghum have competitive advantages over corn and barley since the content of macro-and microelements in it is higher. Thus, grain sorghum is becoming more popular in Russia in recent years^24,25^.

Sorghum is one of the most damaged seeds^24,25^. However, it is an important forage crop to be grown in arid regions of our country, thanks to its resistance to drought and soil tolerance^24^. Sorghum can be used as a raw material for the starch industry. 65 kg of starch can be obtained from 100 kg of sorghum. Sorghum like barley can be successfully used for animal fattening and receiving high weight gain and meat quality. But when feeding pigs with sorghum grain got from 1 ha, twice as much pork as when feeding barley grain can be obtained due to sorghum higher yields^24^.

The experiments used pre-selected sorghum seeds suitable for presowing processing with the number of damaged seeds less than 1.5% of the total weight. The moisture content of the seeds does not exceed the permissible value according to GOST 12041-82, and it didn't exceed 16% in wheat and 13% in sorghum.

Collection of plants was conducted according to the IUCN Policy Statement on Research Involving Species at Risk of Extinction and the Convention on the Trade in Endangered Species of Wild Fauna and Flora. Research was approved by Ethical Committee of Bashkir State Agrarian University, protocol number RBSAU-2020-1124075.

## Results

**Figure 9:**
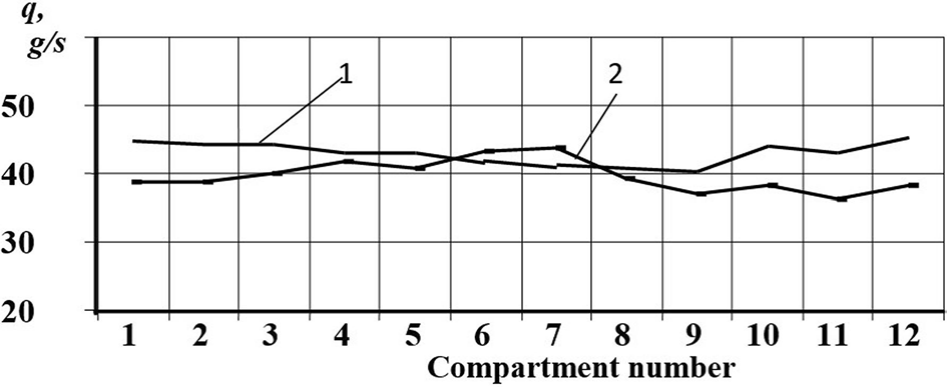
Distribution of seeds over compartments between the blades on the coinciding rotation move. *Source:* author's development.

**Figure 10:**
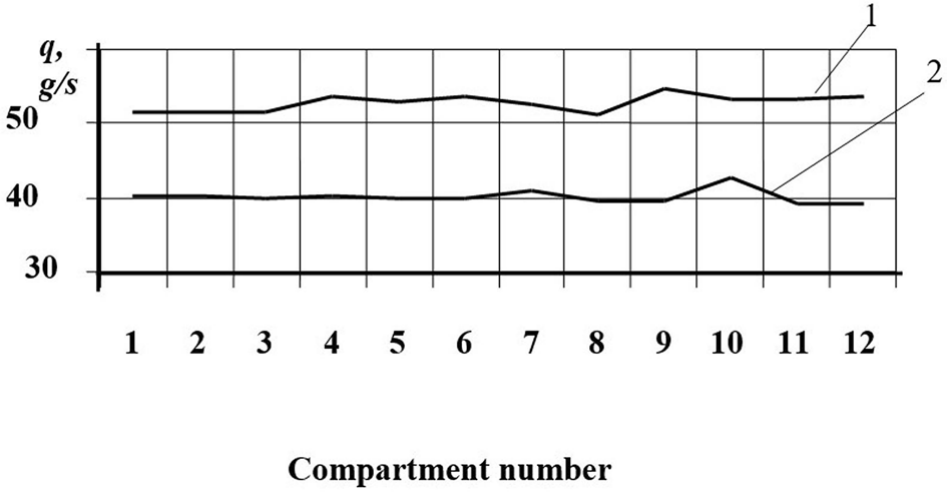
Distribution of seeds over compartments between the blades on the coinciding rotation move. *Source:* author's development.

The experiments allowed us to obtain the dependences of the seed distribution over the compartments between the blades on the coinciding and opposite rotational move of the funnel relative to the centrifugal disk (Figs. 9 and 10). In the figures, curve 1 shows the distribution of winter wheat seeds and curve 2 shows sorghum seeds.

The speed mode of the disk working body rotation during the tests corresponded to the speed mode of the real disk working body of the PS10 type pickling machine.

The coefficients of the dose variation of particles trapped in the catchers were close. Thus, the distribution of winter wheat seeds amounted to − 3.7% and − 5.7% for sorghum seed distribution.

During the counterrotation distribution (Fig. 10), the dose variation coefficients were 4.6% for winter wheat and 5.7% for sorghum grains.

From the graphs shown in Figs. 9 and 10, it can be concluded that the particle distribution is more even with the counterrotation of the director cone and the centrifugal disk.

**Figure 11:**
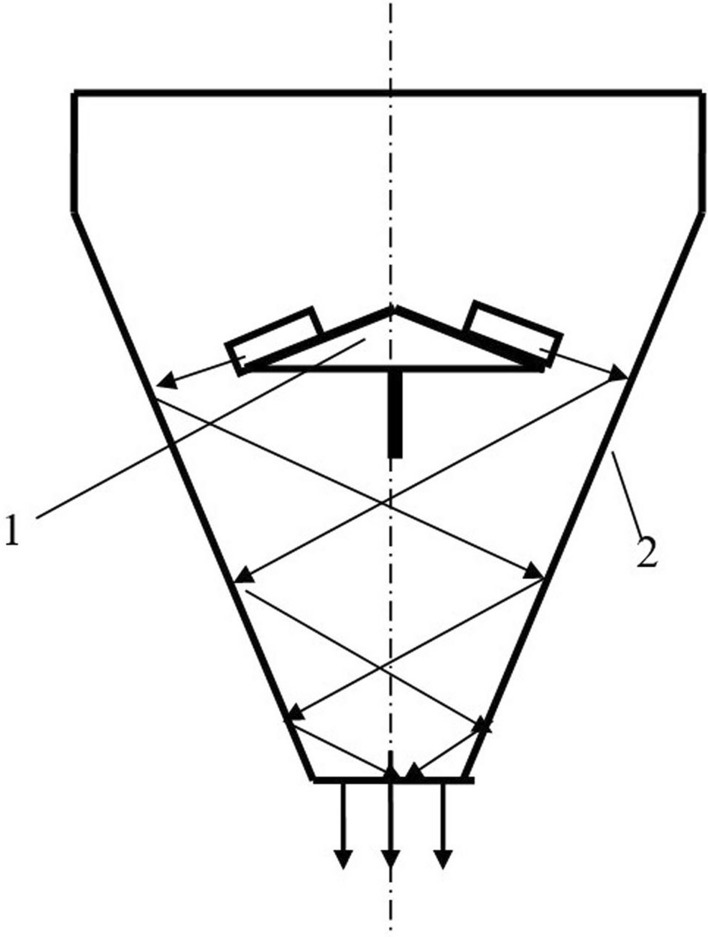
Technological scheme of a centrifugal disk seed treater. *Source:* author's development.

After descent from the blades of the centrifugal disk, the seeds move in the space between disk 1 and reflecting surface 2 at a speed *of V*_*R2*_ (Fig. 11). When reflected from the surface of casing 2, the seeds are hit several times. The first hits are the most dangerous from the point of view of seed damage. With repeated reflections, the impact speed decreases.

A decrease in the velocity of the particles descent from the blades of the centrifugal disk is most often achieved by reducing the angular velocity* ω*_***2***_ of the disk. This way of reducing the seed damage rate is not optimal since the treater productivity decreases with a decrease in the angular velocity of the centrifugal disk.

According to previous studies, elastic materials with shock-absorbing ability proved to be used as reflective surfaces to significantly reduce the degree of seed damage^19,21^.

## Discussion

Studies on the improvement of presowing seed treatment and the development of new technical devices have been conducted in Russia and abroad. Most foreign studies are devoted to introducing biotechnologies and studying the effects of chemical and biological preparations on seeds^2,3,5,7,12,13^.

When studying the influence of various factors on seed sowing qualities, scientists^11,14,16–18,21^ confirmed that mechanical seed damage during the technological processes of crop cultivation is one of the main reasons for seed quality determination.

Unfortunately, there is very little foreign research and few new machines for presowing seed treatment covered by the press since research and development projects in this field of engineering are related to the food security of any country and are not subject to open discussion until they are tested in production and approved by agricultural producers. Among the foreign developers and manufacturers of modern treaters engaged in scientific research and the production of new equipment, the most famous is the Danish company Cimbria and the German companies Petkus and Bayer. Almost all researchers believe that engineers should not use auger working bodies in their machines when developing new equipment to avoid seed damage. Moreover, the treatment speed rate should be decreased. Therefore, drum-type machines are the most promising for presowing seed treatment from the point of view of seed damage prevention. However, when treating seeds, it is necessary to ensure the uniform mixing of seeds with the preparation and the uniform treatment of each seed, which is difficult to achieve when using drum-type treaters. Chamber-type machines with a centrifugal disk working body ensure uniform coating of seeds with the preparation. Another significant disadvantage of existing drum-type machines is their low productivity. Therefore, drum-type treaters and other existing technical means of seed treatment need improvement to obtain new scientific results and create machines with working bodies that ensure three main conditions for high-quality seed treatment: the uniform mixing of seeds with the preparation, the uniform seed coating and their preservation during treatment. Moreover, seed treater developers should achieve the high productivity of technical devices and reduce the energy intensity of the presowing seed treatment. The differentiating feature of this research is the justification of the expediency of further studying the interaction of seeds with the surfaces of working bodies on the example of working bodies of machines used in other technological operations, such as fertilization, and the use of shock-absorbing materials for the manufacture of working bodies of any type^17,18,22,27^.

Table 1 shows the values of the maximum impact force on various materials used in the manufacture of working bodies of agricultural machines^27^. The maximum impact force was determined at the maximum velocity of grain falling in still air *V *_*KR.*_ = 10 m/s.

**Table 1:** Impact force of seeds on surfaces made of various materials.

Material	Velocity of sound shock wave propagation, m/s	Maximum particle impact force (pea seed), H
Steel	5130	4120
Timber	4050	333
Polystyrene	2350	190
Polyethylene	2000	160
Elastomer	160	13

For example, the average destructive load of pea seeds is $F_{pc}^{peas} \approx 302\,{\text{H}}$. Comparing the data in Table 1 with this value, it can be concluded that elastomers are better for use as a shock-absorbing material for manufacturing disc working bodies of machines for pre-sowing seed treatment.

Elastomers have excellent wear resistance, hydrolytic stability and good mechanical characteristics. They are widely used in mechanical engineering, mining and extractive industries and can find application in agricultural engineering.

## Conclusions

The analysis of the interaction of seeds with a centrifugal disk working body in chamber-type treaters and theoretical and experimental studies allowed determination of the conditions necessary to ensure the uniform treatment of seeds with chemical or biological preparations, reducing the impact on seeds and solving the following tasks:

- to establish that the uniform seed treatment and seed damage by disk working bodies is influenced by the following factors: the distance from the axis of rotation of the working body to the center of the feed zone; the shape of the longitudinal and cross-section of the blades; the speed of disk rotation; the speed of seed supply to the blades of the disk; and the direction of the feed speed vector. It has been established that a funnel-shaped director cone providing the seed supply to the working treatment area should be used to reduce the seed impact. In this case, the seed feed rate at the exit of the director cone should coincide with the circumferential speed of the centrifugal disk working body. In addition, the direction of the seed feed rate should coincide with the guide blade setting angle.
- The design parameters and kinematic modes that ensure uniform seed coating with the preparation and reduce seed damage by a centrifugal disk working body have been determined. It has been established that the impact interaction velocity depends on the disk rotation angular velocity, the outer radius of the funnel-shaped director cone, the velocity of the particles escaping from the cone and the direction of the particle velocity vector. The direction of the disk rotation relative to the director cone proved to significantly affect seed damage. It has been established that for the uniform coating of the seeds with the preparation and reducing seed damage by the working body, the particle should be picked up by the blade on the fly. To provide this condition, a formula for determining the angle of the blade setting at the inner edge of the centrifugal disk *Ro*_*Ro*_ was obtained.
- Experimental verification of the obtained theoretical dependencies confirmed the reliability of the theoretical studies. It has been experimentally proven that during the counterrotation of the director cone and the centrifugal disk, it is advisable to install curved blades on the centrifugal disk with an angle determined according to the theoretical dependence *Ro*_*Ro*_. A funnel-shaped cone director and a centrifugal disk with blades made using the obtained theoretical dependencies provided a condition for picking up the particle on the fly for the uniform coating of seeds with the preparation and reducing the seed damage. The distribution of seeds on the developed disc working body is more even for a counter-rotating guide cone and centrifugal disk.

The coefficients of dose variation with counter-rotation of the funnel relative to the centrifugal disk were 4.6% for winter wheat and 5.7% for sorghum grains;

- assessing the impact force of seeds on surfaces of various stiffness, it can be recommended to manufacture disc working bodies from shock-absorbing materials—elastomers that reduce the impact force of seeds by 100 or more times, compared with the impact force on other surfaces.

## Data Availability

Data will be available on request from the corresponding author (Mayya Sukhanova).

## References

[1] Balde S (2021). Effects of seed pretreatment on germination, growth and yield of *Momordica charantia* L. Agric. Sci..

[2] Bezpal'ko VV (2019). Ecologically safe methods for presowing treatment of cereal seeds. Ukr. J. Ecol..

[3] Delkhoshi H, Jalilian J (2012). Effect of presowing seed treatment and spraying of bio-organic nutrient on yield and yield components of maize (*Zea mays* L.). Int. Res. J. Appl. Basic Sci..

[4] Fipke GM (2018). Application of nonselective herbicides in the preharvest of wheat damages seed quality. Am. J. Plant Sci..

[5] Galahitigama GAH, Wathugala DL (2016). Effects of presowing seed treatments on seed germination and salinity tolerance of Rice (*Oryza sativa* L.) seedlings. Int. J. Agron. Agric. Res..

[6] Marks N, Szecówka PS (2010). Impact of variable magnetic field stimulation on growth of aboveground parts of potato plants. Int. Agrophys..

[7] Khasanov E, Kamaletdinov R, Mudarisov S, Shirokov D, Akhunov R (2020). Optimization parameters of the spiral mixing chamber of the device for the sowing seed treatment with biological preparations. Comput. Electron. Agric..

[8] Mugloo JA, Mir NA, Khan PA, Nabi Perray G, Kaisar KN (2016). Effect of different pre-sowing treatments on seed germination of spruce (*Picea smithiana* Wall. Boiss) Seeds under temperate conditions of Kashmir Himalayas, India. Int. J. Curr. Microbiol. Appl. Sci..

[9] Siddique A, Kumar P (2018). Physiological and biochemical basis of pre-sowing soaking seed treatments—An overview. Plant Arch..

[10] Shah T (2017). Influence of pre-sowing seed treatments on germination properties and seedling vigor of wheat. Agric. Vet. Sci..

[11] Laman NA, Aleseychuk GN, Kalatskaia ZN (2006). Modern technology of pre-sowing seed treatment. Sci. Innov..

[12] Nurullin EG (2018). The main directions of improvement of machines for pre-sowing seed treatment. Mach. Equip. Village.

[13] Sukhanova, M. V., Sukhanov, A. V. & Malinovsky, S. V. *Patent 2618106 Russian Federation, IPC A01C 1/06. The Method of Pre-sowing Seed Treatment and the Device for Its Implementation.* Patent holder Sukhanova M. V.-No. 201613101318, application 19.01.2016; publ. 02.05.2017 (2017).

[14] Pehalsky IA, Moskovsky MN (2015). Ways to reduce injury to seeds with sieves. Rural Mech..

[15] Sukhanova MV, Zabrodin VP (2019). Damage to seeds by the working bodies of continuous machines. Int. J. Mech. Prod. Eng. Res. Dev..

[16] Zareiforoush H, Komarizadeh MH, Alizadeh MR (2010). Effects of crop-machine variables on paddy grain damage during handling with an inclined screw auger. Biosyst. Eng..

[17] Tarasenko AP (2019). Reduction of grain damage during post-harvest processing. Bull. Agrarian Sci. Don.

[18] Lobanov VI (2019). Seed grain damage in bucket elevators. Bull. ASAU.

[19] Maryam D, Oskouie B (2011). Study the effect of mechanical damage at processing on soybean seed germination and vigor. J. Agric. Biol. Sci..

[20] Parde SR, Kausal RT, Jayas DS, White ND (2002). Mechanical damage to soybean seed during processing. J. Stored Prod. Res..

[21] Feidengold VB, Beletsky SL (2016). Causes of grain injury and measures to eliminate them. Innov. Technol. Prod. Storage Mater. Values State Needs.

[22] Zabrodin VP (2004). Mechanization of Processes of Adaptive Application of Mineral Fertilizers: Abstract of the Dissertation of the Doctor of Technical Sciences: 05.20.01.

[23] Inss TM, Reese AR (1962). The theory of the centrifugal distributor II. Motion on the disc centrefeed. J. Agric. Eng. Res..

[24] Balakai, S. G. Sorghum is a crop of great opportunities. *Sci. J. Russ. Res. Ins. Land Reclam. Probl.* **1**(5), 83–90 (2021).

[25] Sviridova SA, Negreba ON, Iuzenko YA (2017). Grain sorghum for feed. Inf. Bull. Ministry Agric. Russ. Federation.

[26] Moharam MH, Stephan D, Koch E (2018). Evaluation of plant-derived preparations and microorganisms as seed treatments for control of covered kernel smut of sorghum (*Sporisorium sorghi*). J. Plant Dis. Prot..

[27] Sukhanova MV, Prokhoda AA, Ivanov AN (2019). Experimental determination of the impact force of a surface of various stiffness on seeds. Bull. Agrarian Sci. Don.

